# Recurrence of solitary extramedullary plasmacytoma affecting both optic nerves: a case report

**DOI:** 10.1016/j.ajoc.2025.102438

**Published:** 2025-09-27

**Authors:** Pablo Ballester Dolz, Dimitrios Gkretsis, Marita Andersson Grönlund

**Affiliations:** a Department of Ophthalmology, Örebro University Hospital, Sweden; b Region Västra Götaland, Sahlgrenska University Hospital, Department of Ophthalmology, Mölndal, Sweden; c Department of Ophthalmology, Faculty of Medicine and Health, Örebro University, Sweden; d Department of Clinical Neuroscience, Institute of Neuroscience and Physiology, Sahlgrenska Academy, University of Gothenburg, Sweden

**Keywords:** Plasmacytoma, Optic nerve edema, Multiple myeloma

## Abstract

**Purpose:**

To present a case of atypically located plasmacytoma recurrence with bilateral involvement of the optic nerves.

**Observations:**

A 73-year-old man with diabetes, essential hypertension, and primary hypercholesterolemia presented to our clinic with subjective visual loss in his left eye. The patient had a previous history of solitary extramedullary plasmacytoma in the spinal cord, which was treated with resection surgery at the thoracic T6-T12 levels and curative radiotherapy two years prior. The ophthalmological examination indicated best-corrected visual acuity of 20/50 (0.4 logMAR) in both the right eye (OD) and left eye (OS). Intraocular pressure was normal and fundus examination of the eyes showed edema in the left optic nerve, but no other findings. Magnetic resonance imaging of the brain and orbits showed enhancement in both optic nerves, as in other parts of the central nervous system (CNS).

**Conclusions and importance:**

Plasmacytoma represents a rare form of plasma cell neoplasm. When the condition progresses and affects different sites within the CNS, it is essential to re-evaluate and exclude progression to multiple myeloma and/or other plasma cell neoplasms. Individuals diagnosed with plasmacytoma who complain of visual disturbances should undergo ophthalmologic evaluation.

## 1 Introduction

Plasmacytoma is a rare form of plasma cell dyscrasia that can be found as a solitary tumor either in bones, i.e., solitary bone plasmacytoma (SBP), or in soft tissues, i.e., solitary extramedullary plasmacytoma (SEP). When a plasma cell neoplasm proliferates and leads to multiple lytic bone lesions, or if it is associated with anemia, hypercalcemia, and/or kidney impairment, it is classified as multiple myeloma (MM).^1^

SBP is the predominant form of plasmacytoma, with an incidence rate 40 % higher than that of the SEP subtype.^2^ SBP represents a small percentage of all plasma cell disorders (2–6 %)^3^^,^^4^ and has an estimated worldwide incidence of 0.34 per 100,000 persons/year.^4^ The incidence of SEP is even lower, affecting around 0.10 per 100,000 persons/year,^4^ and the average age at which SEP is diagnosed is 55–60 years, with males constituting around two-thirds of the patients.^4^

The primary treatment for patients with plasmacytomas (SBP and SEP) is localized radiation therapy.^5^ In certain cases, surgical resection is performed as part of the diagnostic process or if patients experience cord compression or bone instability. The use of adjuvant or prophylactic systemic therapy does not seem to significantly affect the relapse rate or disease-free survival.^4^

Despite effective management with radiotherapy, either alone or in combination with surgery, 40–50 % of patients diagnosed with SBP will develop MM within ten years.^3^^,^^6^ In contrast, the rate of progression to MM is lower in patients with SEP, at only 10–15 %.^6^ The 10-year survival rate of patients diagnosed with SEP is 22–77 %.^7^

We present a case of SEP recurrence affecting both optic nerves as well as other central nervous system (CNS) structures.

## 2 Case report

A 73-year-old male patient with diabetes, essential hypertension, and primary hypercholesterolemia presented to the outpatient clinic of the Department of Ophthalmology at Örebro University Hospital, Sweden, with a one-week history of progressive vision loss in the left eye (OS). Additionally, the patient reported a decline in strength in his right leg over the previous approximately two months.

According to the patient's medical records, surgery was performed three years prior to relieve spinal cord compression at levels T6–T12, which was caused by a tumor. Initially, the tumor was diagnosed as a T-cell lymphoma, and the patient received one dose of chemotherapy with cyclophosphamide, hydroxydaunorubicin, vincristine sulfate (Oncovin®) and prednisone (CHOP). However, after a multidisciplinary discussion involving hematologists, oncologists, and pathologists, the biopsy results were re-evaluated, leading to a change in diagnosis to SEP. Subsequently, the patient underwent a curative radiotherapy treatment with a total dose of 50 Gy.

**Fig. 1:**
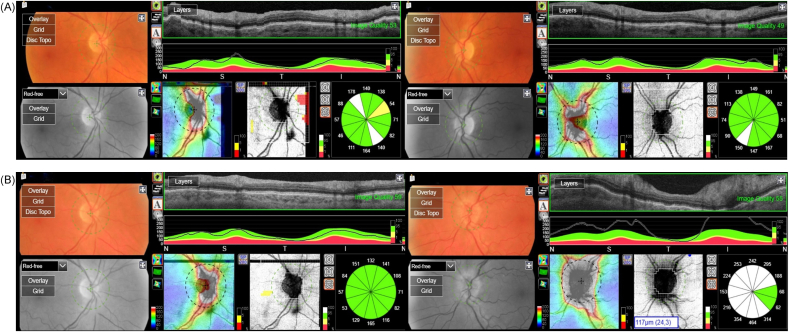
Optic nerve optical coherence tomography (OCT) in a 73-year-old man with history of solitary extramedullary plasmacytoma (SEP). **(A)** OCT performed 2 years prior to presentation, after the treatment of SEP with surgery and radiotherapy (50 Gy) to the spinal cord, shows normal optic nerves with no signs of swelling. **(B)** OCT performed at presentation, 3 years after the diagnosis of SEP, shows a normal optic nerve in the right eye (OD) and increased thickness (i.e. optic disc swelling) in the left eye (OS).

At the time of diagnosis three years prior, the blood tests detected an increase in kappa-free light chain levels and slightly decreased serum albumin. Serum calcium, serum creatinine, venous lactate dehydrogenase, C-reactive protein, erythrocyte sedimentation rate (ESR) and serum free light chain (FLC) ratio were normal. Urinalysis showed increased rates of protein and slightly increased levels of kappa and lambda light chains but FLC ratio in urine was normal. No signs of anemia or reduced renal function were found. Bone marrow biopsy showed no signs of neoplastic plasma cell involvement. Three months later, serum protein electrophoresis test showed the presence of monoclonal immunoglobulin (IgG) kappa (M protein), which remained stable during the follow-up period at around 1 g/L. Follow-up examinations with computed tomography (CT) and magnetic resonance imaging (MRI) during the first year after treatment did not reveal any recurrence of the disease. Optical coherence tomography (OCT) images taken 2 years prior after the treatment of SEP, showed a normal optic nerve in both eyes (Fig. 1A).

On initial presentation to our clinic, the patient's best-corrected visual acuity (BCVA) was 20/50 (0.4 logMAR) in both OD and OS. The patient denied experiencing double vision or pain when moving his eyes. No relative afferent pupillary defect was found. The extraocular movements were full in both eyes. Color vision testing with Ishihara pseudoisochromatic plates and Sahlgren's saturation test (SST),^8^ with the latter detecting acquired dyschromatopsia, yielded abnormal results in both eyes. The intraocular pressure was normal in both eyes. Slit-lamp examination of the anterior segment did not show any signs of inflammation in either eye. Senile nuclear cataract was present in both eyes. Dilated fundus examination was normal in the OD but revealed optic nerve edema in the OS. OCT analysis with DRI-OCT Triton® (TOPCON®, Tokyo, Japan) showed an increased thickness of the retinal nerve fiber layer (RNFL), confirming edema in the head of the left optic nerve (Fig. 1B).

**Fig. 2:**
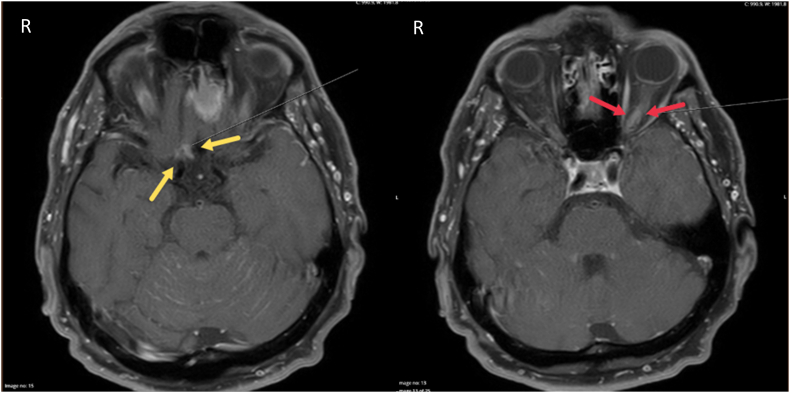
Magnetic resonance imaging (MRI) of the brain and orbits in a 73-year-old man with history of solitary extramedullary plasmacytoma (SEP), performed at presentation, 3 years after the diagnosis of SEP, shows enhancement in both the right (yellow arrows) and left (red arrows) optic nerves, suggesting the recurrence of the previously diagnosed SEP that had been treated with surgery and curative radiotherapy. (For interpretation of the references to colour in this figure legend, the reader is referred to the Web version of this article.)

MRI of the brain and orbits showed a slight volume increase enhancement in the left optic nerve and discrete enhancement in the right optic nerve adjacent to the chiasm (Fig. 2). Additionally, multiple contrast-enhancing lesions of several sites (i.e. corpus callosum, cerebellum and two areas adjacent to both olfactory nerves) were observed, suggesting leptomeningeal tumor spread. A complementary CT myelography showed no MM signs in the skeleton. Unexpectedly, blood tests showed no visible M component in the serum, and lumbar puncture showed normal levels of proteins and no evidence of intrathecal IgG production. Hence, MM and other plasma cell neoplasia were ruled out. The patient was treated with palliative radiotherapy of the brain and orbits (4Gy × 5). Oral pomalidomide-dexamethasone was given as an adjuvant treatment (Imovid®, 4 mg, 21-day cycle, the first 14 days 4 mg/day followed by a 7-day break, in combination with dexamethasone 20 mg on days 1, 8, and 15 of the 21-day cycle).

**Fig. 3:**
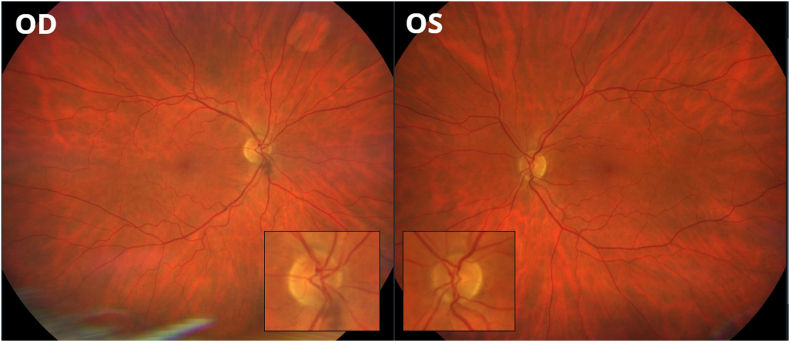
Fundus photography in a 73-year-old man with history of solitary extramedullary plasmacytoma (SEP), performed 10 months after the treatment of the recurrence of SEP with palliative radiotherapy of the brain and orbits (4Gy × 5) and oral pomalidomide-dexamethasone, shows total regression of optic nerve edema in the left eye (OS).

MRI images two months post treatment with radiotherapy and systemic medication showed regression of all contrast-enhancing changes in both the intracranial and intraspinal regions. No further eye examinations were performed as the patient's general health worsened. However, two telephone follow-ups were conducted, and the patient did not report any visual symptoms during the six months following the diagnosis of optic nerve edema. An eye fundus photograph was taken as part of a diabetic retinopathy screening ten months after the initiation of palliative care, at a tertiary center, and no optic nerve edema was observed in either eye (Fig. 3). The last telephone contact with the patient was made 1 year after presentation, and no visual symptoms were reported; however, severe back pain was the main issue for the patient.

## 3 Discussion

This case report describes a patient with a relapse of SEP affecting both optic nerves and other CNS structures. CNS involvement in this type of plasma cell neoplasm is a rare complication reported in under 1 % of patients.^9^^,^^10^

Plasmacytomas occurring in the ocular and adnexal tissue are also infrequent and can manifest either as primary SEP or as secondary plasmacytoma in the context of MM.^11^ When the eyes or adnexa are affected by plasmacytomas, orbital tissue involvement is more common than ocular involvement.^9^^,^^12^

Involvement of the optic nerve in SEP is extremely rare, with only a few cases having been reported in the literature.^12^ In these cases, the optic nerve damage can manifest as ischemia resulting from hyperviscosity syndrome, direct infiltration, or compression caused by the plasmacytoma.^13^ Here, we describe a case of bilateral optic nerve involvement in SEP.

In cases in which metastatic spread of plasmacytomas (i.e., SBP and SEP) occurs, further investigations with a new bone marrow biopsy and/or other diagnostic imaging testing are compulsory to rule out that the patient has experienced progression to MM. When choosing the imaging technique, using the same imaging method for both diagnosis and monitoring treatment response is advised,^14^ and positron emission tomography (PET)/CT is generally preferred to MRI due to its superior sensitivity.^15^

Consensus is lacking on the approach to treating relapse of SEP.^16^ Individualized assessment is necessary to determine whether SEP has evolved to MM or whether it is a local recurrence. In instances of relapse at a different site, systemic disease should be highly suspected. There are different approaches when a local relapse of plasmacytoma occurs: if the lesion occurs at the same location after surgery, radiotherapy is the preferred treatment; if the lesion occurs at the same location after radiotherapy, surgical resection or additional radiotherapy can be a reasonable approach. If relapse occurs at a different location, systemic disease should be highly suspected and systemic treatment may be necessary.^16^

## 4 Conclusion

Plasmacytoma is an uncommon type of plasma cell neoplasm. In instances in which the disease spreads and affects different structures within the CNS, it is crucial to rule out systemic diseases such as MM and other plasma cell neoplasms. Patients with SEP who report visual disturbances should undergo a comprehensive ophthalmologic examination.

## CRediT authorship contribution statement

**Pablo Ballester Dolz:** Writing – original draft, Resources, Investigation, Data curation, Conceptualization. **Dimitrios Gkretsis:** Writing – review & editing, Resources, Data curation. **Marita Andersson Grönlund:** Writing – review & editing, Validation, Supervision.

## Patient consent

Written informed consent was obtained from the patient for publication of the details of his medical case and any accompanying images.

## Authorship

All authors attest that they meet the current ICMJE criteria for authorship.

## Claims of priority

A literature review was conducted on 04/17/2024, using PubMed and Google Scholar with the search terms “extramedullary plasmacytoma” AND “CNS.” No prior reports were found describing recurrence of plasmacytoma involving both optic nerves.

There are publications that report plasmacytomas in the CNS and in the optic nerve but not bilaterally.

## Funding

No funding or grant support.

## Declaration of competing interest

The authors declare that they have no known competing financial interests or personal relationships that could have appeared to influence the work reported in this paper.

## References

[1] Rajkumar S.V., Dimopoulos M.A., Palumbo A. (Nov 2014). International myeloma working group updated criteria for the diagnosis of multiple myeloma. Lancet Oncol.

[2] Grammatico S., Scalzulli E., Petrucci M.T. (2017). Solitary plasmacytoma. Mediterr J Hematol Infect Dis.

[3] Dimopoulos M.A., Moulopoulos L.A., Maniatis A., Alexanian R. (2000). Solitary plasmacytoma of bone and asymptomatic multiple myeloma. Blood.

[4] Dores G.M., Landgren O., McGlynn K.A., Curtis R.E., Linet M.S., Devesa S.S. (2009). Plasmacytoma of bone, extramedullary plasmacytoma, and multiple myeloma: incidence and survival in the United States, 1992-2004. Br J Haematol.

[5] Goyal G., Bartley A.C., Funni S. (Jun 2018). Treatment approaches and outcomes in plasmacytomas: analysis using a national dataset. Leukemia.

[6] Curry J., O'Steen L., Morris C.G., Kirwan J.M., Mendenhall W.M. (Oct 2020). Long-term outcomes after definitive radiation therapy for solitary plasmacytoma. Am J Clin Oncol.

[7] Shen X., Zhang L., Wang J., Chen L., Liu S., Zhang R. (2022). Survival trends and prognostic factors for patients with extramedullary plasmacytoma: a population-based study. Front Oncol.

[8] Lindblom B., Wikholm M., Frisen L. (1988). Sahlgren's saturation test for acquired dyschromatopsia: increased lightness enhances sensitivity. Graefes Arch Clin Exp Ophthalmol.

[9] Yeung S.N., Paton K.E., Dorovini-Zis K., Chew J.B., White V.A. (Mar 2008). Histopathologic features of multiple myeloma involving the optic nerves. J Neuro Ophthalmol.

[10] Franzon C.R., Wagner A.O.M., Lopes A.C.W., Bona D.G., Schmitz T.B. (2021). MM-340: plasma cell neoplasm in the central nervous system: a case report. Clin Lymphoma Myeloma Leuk.

[11] Adkins J.W., Shields J.A., Shields C.L., Eagle R.C., Flanagan J.C., Campanella P.C. (1996). Plasmacytoma of the eye and orbit. Int Ophthalmol.

[12] Sandor K.P., Micieli J.A., Peragallo J.H. (Jan-Mar 2021). Optic nerve head plasmacytoma as a manifestation of multiple myeloma. Taiwan J Ophthalmol.

[13] Maini R., Macewen C.J. (May 1997). Intracranial plasmacytoma presenting with optic nerve compression. Br J Ophthalmol.

[14] Caers J., Paiva B., Zamagni E. (Jan 16 2018). Diagnosis, treatment, and response assessment in solitary plasmacytoma: updated recommendations from a european expert panel. J Hematol Oncol.

[15] Fouquet G., Guidez S., Herbaux C. (Jun 15 2014). Impact of initial FDG-PET/CT and serum-free light chain on transformation of conventionally defined solitary plasmacytoma to multiple myeloma. Clin Cancer Res.

[16] Mateos M.-V., Connor R.F. (2024). Uptodate.

